# Fetal Urinary Cystatin C, NGAL and Beta-2-Microglobulin as Predictors of Postnatal Renal Function Impairment and Death in Fetuses with Lower Urinary Tract Obstruction

**DOI:** 10.3390/jcm15052056

**Published:** 2026-03-08

**Authors:** Małgorzata Stańczyk, Krzysztof Badura, Ayaana Ibshaan, Katarzyna Fortecka-Piestrzeniewicz, Iwona Maroszyńska, Tomasz Talar, Dariusz Olejniczak, Michał Podgórski, Jolanta Romak, Zuzanna Gaj, Krzysztof Szaflik, Piotr Kaczmarek, Marcin Tkaczyk

**Affiliations:** 1 Department of Pediatrics, Nephrology and Immunology, Polish Mother’s Memorial Hospital Research Institute, 93-338 Łódź, Poland; 2 Department of Pediatrics, Nephrology and Immunology, Medical University of Lodz, 90-419 Łódź, Poland; ayaana.ibshaan@stud.umed.lodz.pl; 3 Faculty of Medicine, Medical University of Lodz, 90-419 Łódź, Poland; 4 Department of Intensive Therapy and Congenital Malformations of Newborns and Infants, Polish Mother’s Memorial Hospital Research Institute, 93-338 Łódź, Poland; katarzyna.fortecka-piestrzeniewicz@iczmp.edu.pl (K.F.-P.); iwona.maroszynska@iczmp.edu.pl (I.M.); 5 Department of Neonatology and Intensive Care, Polish Mother’s Memorial Hospital Research Institute, 93-338 Łódź, Poland; tomasz.talar@iczmp.edu.pl; 6 Department of Surgery and Urology, Polish Mother’s Memorial Hospital Research Institute, 93-338 Łódź, Poland; dariusz.olejniczak@iczmp.edu.pl; 7 Department of Diagnostic Imaging, Polish Mother’s Memorial Hospital Research Institute, 93-338 Łódź, Poland; michal.podgorski@iczmp.edu.pl; 8 Medical Laboratory Diagnostic Centre, Polish Mother’s Memorial Hospital Research Institute, 93-338 Łódź, Poland; jolanta.romak@iczmp.edu.pl (J.R.); zuzanna.gaj@iczmp.edu.pl (Z.G.); 9 Department of Gynecology, Fertility and Fetal Therapy, Polish Mother’s Memorial Hospital Research Institute, 93-338 Łódź, Polandpiotr.kaczmarek@iczmp.edu.pl (P.K.)

**Keywords:** lower urinary tract obstruction, prediction, prenatal, fetal urine analysis, rare disease, cystatin C, NGAL, beta-2-microglobulin

## Abstract

**Background/Objectives**: Fetal lower urinary tract obstruction (LUTO) is a rare congenital anomaly that often leads to pulmonary hypoplasia and kidney dysfunction, which contribute to increased mortality. Prenatal estimation of the severity of LUTO is challenging due to the lack of specific diagnostic tools, which may guide clinical decisions. The aim of this analysis was to assess the role of fetal urinary concentrations of neutrophil gelatinase-associated lipocalin (NGAL), β2-microglobulin (B2M) and Cystatin C (CysC) in the prediction of unfavorable outcomes, such as postnatal renal dysfunction and death, among LUTO patients. **Methods**: A total of 38 women carrying fetuses with suspected LUTO (based on ultrasound features) were included in the study. Fetal urine was collected from the bladder of the fetus under ultrasound guidance, and measurements of NGAL, CysC and B2M were performed using an enzyme-linked immunosorbent assay. We analyzed the role of NGAL, CysC and B2M in the prediction of renal dysfunction or death within 30 days after birth. **Results**: Fetal urinary NGAL, CysC and B2M corrected for fetal urinary creatinine (FuCr) were significant predictors of impaired postnatal renal function or death within 30 days after birth. AUCs of ROC curves for NGAL/FuCr, CysC/FuCr and B2M/FuCr as predictors of renal dysfunction or death within 30 days after birth were: 0.793 (95% CI: 0.614–0.972, *p* = 0.001), 0.857 (95% CI: 0.7–1.0, *p* < 0.0001), 0.764 (95% CI: 0.562–0.966, *p* = 0.01), respectively. Among assessed biomarkers, only CysC/FuCr corrected for creatinine (*p* = 0.02) was associated with decreased eGFR on day 30 of postnatal life, whereas NGAL (*p* = 0.07) and B2M (*p* = 0.12) were not. AUCs of ROC curves for NGAL/FuCr, CysC/FuCr and B2M/FuCr as predictors of renal dysfunction on day 30 after birth were: 0.756 (95% CI: 0.535–0.976, *p* = 0.02), 0.833 (95% CI: 0.649–1.0, *p* = 0.0004), 0.722 (95% CI: 0.482–0.963, *p* = 0.07), respectively. **Conclusions**: Fetal urinary NGAL, CysC and B2M may constitute a promising tool in early prediction of impaired renal function and mortality in fetuses with LUTO. Accurate prediction of renal function decline after birth is crucial for proper pre- and postnatal counseling and may support prenatal intervention decision making. Further studies are required to establish the role of the studied biomarkers in the prediction of adverse outcomes.

## 1. Introduction

Fetal lower urinary tract obstruction (LUTO) is a rare congenital anomaly with an incidence of 1 per 5000–25,000 pregnancies. Although the causes may vary, the most prevalent cause of LUTO is posterior urethral valve (PUV) in males, whereas urethral atresia is the leading cause of LUTO in females. Over 10% of LUTO cases are associated with the trisomy 13, 18, and 21 chromosomes [1].

LUTO results in variable clinical sequelae, including oligohydramnios and its complications, such as secondary pulmonary hypoplasia, which contribute to increased fetal mortality. Kidney dysfunction in patients with LUTO appears to have a multifactorial and complex etiology. First, impaired urine outflow increases hydrostatic pressure within the urinary tract and leads to vesicoureteral reflux, resulting in renal pelvis dilation and parenchymal dysplasia. Second, an increasing role of inflammation contributing to renal failure has been reported. In LUTO, inflammation constitutes a response to impaired urine outflow [2].

Typical sonographic features of fetuses with LUTO include an enlarged bladder (megacystis) with a wall thickness > 2 mm; unilateral or bilateral hydronephrosis, ureterectasis, and caliectasis (seen in 40–50% of cases); hyperechoic kidneys that are small for gestational age; and oligohydramnios or anhydramnios depending on the severity [1,3]. Prenatal LUTO has classically been suspected based on three ultrasonographic findings: megacystis, dilated posterior urethra (known as the ‘keyhole sign’) and hydronephrosis [3].

It has been suggested that biochemical analysis of fetal urine, fetal blood, and amniotic fluid could provide valuable insights into the prognosis for renal function [4]. Fetal blood and/or urine biomarkers, including sodium, chloride, calcium, osmolarity and β2-microglobulin (B2M), have been proposed as predictors of postnatal kidney function in fetuses with LUTO [3]. B2M, a low-molecular-weight protein, is entirely filtered, reabsorbed and degraded by the nephron; serum levels rise when glomerular filtration is impaired, while urinary levels increase when tubular reabsorption is affected [5]. Recent studies have shown increased fetal urinary levels of B2M in fetuses with LUTO. Moreover, fetal urinary B2M levels correlate with inflammatory biomarkers, which may pathophysiologically contribute to renal dysfunction and are associated with unfavorable outcomes [2,6,7].

Neutrophil gelatinase-associated lipocalin (NGAL), also known as lipocalin-2, a protein encoded by the *LCN2* gene, is another urinary biomarker expressed naturally in cytoplasmic granules of neutrophils. It has been identified in several cells in different organ systems, including the kidneys. Previous studies have reported the potential use of NGAL as a predictor of renal (especially tubular) injury and hydronephrosis [8].

Cystatin C (CysC) is a non-glycosylated protein produced by all human nucleated cells, which can be used for eGFR estimation when measured in serum. When compared to serum creatinine, cystatin C does not overestimate renal function in specific clinical conditions (e.g., liver cirrhosis; muscle mass variability) [9]. Under normal conditions, only small amounts of CysC can be found in urine, as it undergoes metabolism in the proximal tubules. Damage to the proximal tubules, which is reported to occur in LUTO, impairs catabolism and may result in increased CysC urinary output [10].

Prenatal diagnosis, severity assessment, and optimal management of LUTO are challenging, owing to the lack of specific diagnostic features that can guide clinical approaches and decisions [3]. In this study, we aim to focus on biomarkers with unestablished significance in the prediction of renal dysfunction and prognosis in LUTO patients, such as fetal urinary B2M, NGAL and CysC. The aim of this analysis was to assess the role of fetal urinary concentrations of NGAL, B2M and CysC in the prediction of unfavorable outcomes such as postnatal renal dysfunction and death among LUTO patients.

## 2. Materials and Methods

### 2.1. Clinical Data and Fetal Urine Sample Collection

A total of 38 women carrying fetuses with suspected LUTO were included in the study. Patients were recruited between January 2016 and September 2017 at a high-volume tertiary gynecological and pediatric center (5634 births between January 2016 and September 2017). The suspicion of LUTO was made based on prenatal ultrasound findings, such as megabladder and/or anhydramnios/oligohydramnios. Prenatal ultrasound was performed in each patient at the time of fetal urine sampling. The study endpoints were: prenatal death; all-cause mortality of live-born neonates with impaired renal function, defined as estimated (based on Schwartz formula); glomerular filtration rate < 20 mL/min/1.73 m^2^; and composite endpoint, including postnatal renal dysfunction on postnatal day 30 or death within 30 days after birth. For live-born neonates, renal function was assessed on postnatal day 4 and day 30. Renal function assessment was based on serum creatinine (sCr) measurement and glomerular filtration rate estimation based on the Schwartz formula (eGFR = [0.413 × height (cm)]/sCr (g/dL)) [11]. Patients with confirmed megabladder (irrespective of hydronephrosis) and reduced amniotic fluid (AFI ≤ 8 mm) were offered a vesicoamniotic shunting [12]. Full patients’ characteristics are available in Table 1.

The study was performed in accordance with the Declaration of Helsinki [13] and with Good Clinical Practice guidelines. The research was approved by the local (Lodz, Poland) ethics committee (No. 1/2016), and informed consent was obtained from each participant.

### 2.2. Fetal Urinary Biomarkers Analysis

Fetal urine was collected from the bladder of the fetus under ultrasound guidance and immediately frozen to a temperature of −80 degrees Celsius. NGAL, CysC and B2M were measured using enzyme-linked immunosorbent assay (Quantikine ELISA kits, R&D Systems, Minneapolis, MN, USA). Fetal urine sodium and chloride concentrations, osmolality, B2M, NGAL and CysC outputs were measured in 38 fetuses included in the study and corrected for creatinine.

Analyses were conducted to assess the following associations for the evaluated biomarkers: (a) differences in fetal urinary concentrations; (b) assessment of their performance in the prediction of postnatal death, kidney dysfunction and composite endpoint (kidney dysfunction or death); (c) estimation of optimal cutoffs for the presented biomarkers, with calculation of sensitivity and specificity; (d) comparison of the predictive performance of selected biomarkers and their previously established thresholds with thresholds estimated in our study; (e) correlation between selected biomarkers and postnatal renal function including eGFR.

### 2.3. Statistical Analysis

Statistical analysis was performed with the use of the Statistica 13.3 software (Tibco, Palo Alto, CA, USA). All tests were considered significant at a *p*-value below 0.05. Data distribution was verified using the Shapiro–Wilk test. Categorical variables were presented as numbers and percentages, while numerical variables were presented as medians with interquartile range (Q1–Q3 [IQR]) based on the normality of their distribution. Categorical variables were compared with respect to the data using Fisher’s exact test and the χ2 test, whereas comparison of continuous variables between groups was performed using the t-Student test, the U Mann–Whitney test, the ANOVA test with the post hoc Turkey’s test, or the Kruskal-Wallis test with the post hoc U Mann-Whitney test with Bonferroni correction, when appropriate. Predictive performance was assessed by calculating the area under the receiver operating characteristic curve (AUC) and 95% confidence interval (DeLong method). Thresholds for specific biomarkers were estimated using the Youden index, which was followed by specificity and sensitivity estimation. Odds ratios for specific biomarkers were estimated using univariable logistic regression. Associations between continuous variables were visualized using scatter plots and quantified as Pearson’s correlation coefficient (r).

## 3. Results

### 3.1. Short-Term Outcomes in Fetuses with LUTO

**Figure 1:**
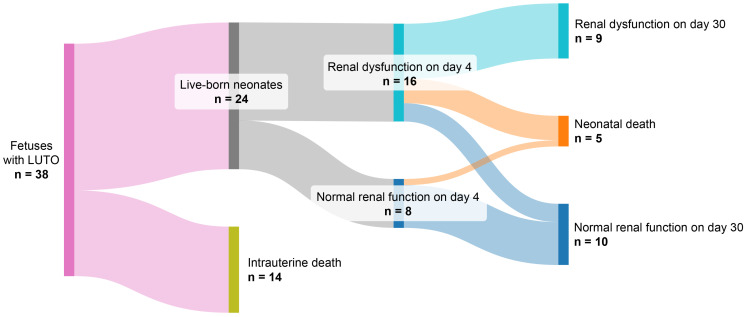
Sankey plot presenting evolution of renal function/survival within the studied cohort.

Of 38 fetuses, intrauterine death occurred in 14 (36.84%). In total, 16 out of 24 neonates had renal dysfunction on day 4. In nine of them, renal dysfunction persisted on day 30; four neonates with renal dysfunction on day 4 died within 30 days postnatally, whereas only two neonates had recovery of renal function. None of the neonates who had normal renal function on day 4 developed renal dysfunction on day 30, and only one neonate with previous normal renal function died. Figure 1 illustrates renal function and adverse clinical outcome development in the observed fetuses and neonates presented as a Sankey plot. Table 2 presents the baseline characteristics of live-born neonates.

### 3.2. NGAL, Cystatin C and β2-Microglobulin in Prediction of Postnatal Renal Dysfunction and Mortality in Fetuses with LUTO

**Figure 2:**
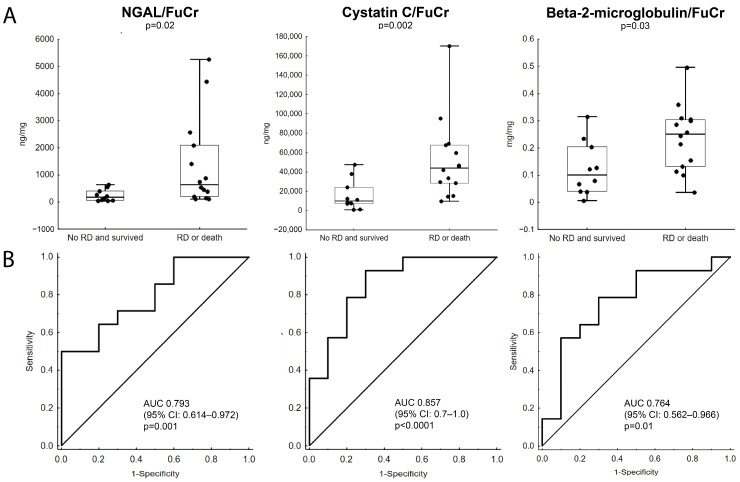
Fetal urine NGAL/FuCr, cystatin C/FuCr and β2-microglobuline/FuCr concentrations in fetuses with lower urinary tract obstruction (LUTO), renal dysfunction and postnatal deaths. (**A**) NGAL, cystatin C and β2-microglobulin concentrations are significantly higher in fetuses who later develop renal dysfunction, defined as decreased eGFR on the 30th day of extrauterine life or death within 30 days after birth. Horizontal line represents median, box represents IQR Q1–Q3, whiskers represent minimum to maximum range. (**B**) receiver operator characteristic curves for NGAL, cystatin C and β2-microglobulin as predictors of decreased eGFR assessed on the 30th day of postnatal life or death within 30 days after death. AUC—area under the curve; CI—confidence interval; eGFR—estimated glomerular filtration rate based on Schwartz formula; FuCr—fetal urinary creatinine; IQR—interquartile range; NGAL—neutrophil gelatinase-associated lipocalin; RD—renal dysfunction.

NGAL/FuCr (*p* = 0.02), CysC/FuCr (*p* = 0.002) and B2M/FuCr (*p* = 0.03) were significantly higher in fetuses with decreased eGFR on postnatal day 30 or in those who died within 30 days after birth when compared to fetuses without renal dysfunction (Figure 2A). AUCs of ROC curves for NGAL/FuCr, CysC/FuCr and β2-microglobin/FuCr as predictors of renal dysfunction or death within 30 days after birth were: 0.793 (95% CI: 0.614–0.972, *p* = 0.001), 0.857 (95% CI: 0.7–1.0, *p* < 0.0001), 0.764 (95% CI: 0.562–0.966, *p* = 0.01), respectively (Figure 2B). No such differences have been observed for fetal urinary sodium, chloride, or osmolality.

Comparable results were obtained for biomarkers without correction. Fetuses who developed renal dysfunction within the first 30 days of postnatal life or died within 30 days had significantly higher NGAL (median: 24.68 vs. 7.78 ng/mL [IQR: 7.37–67.78 vs. 2.01–19.98 ng/mL], *p* = 0.02), CysC (median: 1364.33 vs. 412.64 ng/mL [IQR: 884.76–2778.56 vs. 223.00–798.84 ng/mL], *p* = 0.002) and B2M (median: 9.14–3.58 mg/mL [IQR: 4.08–11.76 vs. 2.13–7.21 mg/mL], *p* = 0.02) when compared to those who survived 30 days without renal dysfunction. Areas under the curve (AUCs) of receiver operator characteristic curves (ROC) for NGAL, CysC and B2M as predictors of renal dysfunction on postnatal day 30 or death within 30 days postnatally were as follows: 0.786 (95% CI: 0.604–0.968, *p* = 0.002), 0.879 (95% CI: 0.733–1.0, *p* < 0.0001), 0.779 (95% CI: 0.592–0.965, *p* = 0.003), respectively.

Higher concentrations of CysC (*p* = 0.04) and B2M (*p* = 0.047) were associated with an increased risk of renal dysfunction or death within 30 days after birth, whereas NGAL (*p* = 0.12) did not reach statistical significance. ORs for NGAL, CysC and B2M were as follows: 1.072 (95% CI: 0.982–1.171), 1.002 (95% CI: 1.002–1.004) and 1.27 (95% CI: 1.003–1.618). Based on the ROC curve, exploratory descriptive data, including thresholds for NGAL, CysC, B2M, and their corrected values, have been presented in Table 3. Moreover, the table includes conventional biomarkers utilized in the prediction of renal dysfunction. It should be noted that, due to several limitations of the study (see limitations in Section 4, Discussion), the results are not applicable in clinical practice. Comparison between neonates with renal dysfunction or death and those that survived without renal dysfunction is presented in Table S1.

**Figure 3:**
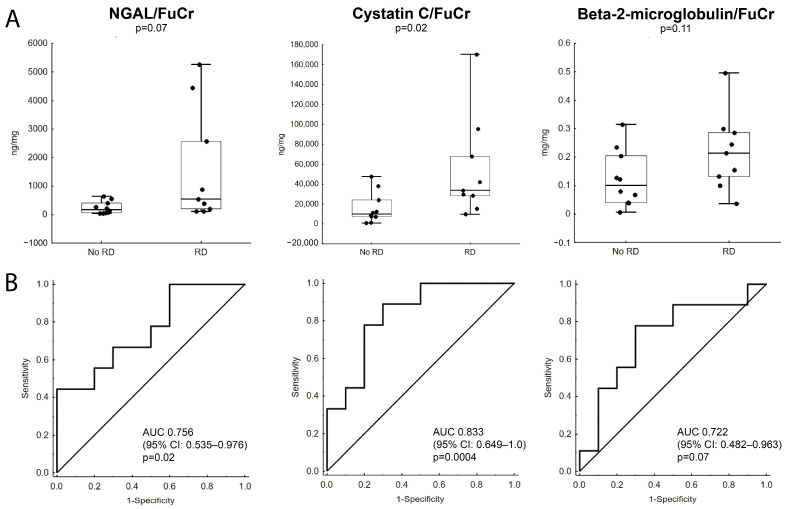
Fetal urine NGAL/FuCr, cystatin C/FuCr and β2-microglobuline/FuCr in fetuses with lower urinary tract obstruction (LUTO) and eGFR assessed on the 30th day of postnatal life. (**A**) Cystatin C/FuCr ratios are significantly higher in fetuses who later develop renal dysfunction, defined as decreased eGFR on the 30th day of extrauterine life, whereas NGAL/FuCr and β2-microglobulin/FuCr are not. Horizontal line represents median, box represents IQR Q1–Q3, whiskers represent minimum to maximum range. (**B**) Receiver operator characteristic curves for NGAL/FuCr, cystatin C/FuCr and β2-microglobulin/FuCr as predictors of decreased eGFR assessed on the 30th day of postnatal life. AUC—area under the curve; CI—confidence interval; eGFR—estimated glomerular filtration rate based on Schwartz formula; FuCr—fetal urinary creatinine; IQR—interquartile range; NGAL—neutrophil gelatinase-associated lipocalin; RD—renal dysfunction;.

**Figure 4:**
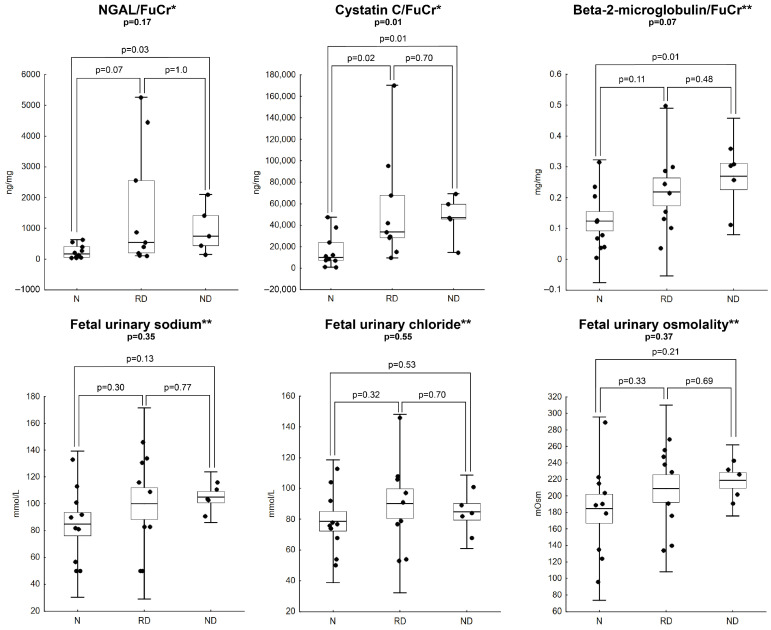
Comparison of fetal urinary concentration among neonates who survived 30 days without renal dysfunction (N), had renal dysfunction on day 30 postnatally (RD), and died within 30 days postnatally (ND). General *p*-value represents the result of a comparison using a one-way ANOVA test for normal distribution and a Kruskal–Wallis test for other than normal distribution. *p*-value for each comparison of two subgroups was obtained using the t-Student test for variables with normal distribution and the U Mann–Whitney test for non-normal distribution. * Other than normal distribution: horizontal line represents median, box represents IQR Q1–Q3, whiskers represent minimum to maximum range; ** normal distribution: horizontal line represents average, box represents standard error, whiskers represent 2 standard deviations.

Only CysC/FuCr (*p* = 0.02) was associated with decreased eGFR on day 30 of postnatal life, whereas NGAL/FuCr (*p* = 0.07) and B2M/FuCr (*p* = 0.12) were not (Figure 3A). AUCs of ROC curves for NGAL/FuCr, CysC/FuCr and B2M/FuCr as predictors of renal dysfunction on day 30 after birth were: 0.756 (95% CI: 0.535–0.976, *p* = 0.02), 0.833 (95% CI: 0.649–1.0, *p* = 0.0004), 0.722 (95% CI: 0.482–0.963, *p* = 0.07), respectively (Figure 3B). Significantly higher Cystatin C/FuCr (*p* = 0.01) and NGAL/FuCr (*p* = 0.03) were also observed among neonates who died within 30 days after birth when compared to those who survived without developing renal dysfunction. All comparisons between subgroups of live-born neonates (neonates who died within 30 days after birth [ND], neonates who developed renal dysfunction within 30 days after birth [RD], and neonates who survived without renal dysfunction [N]) are presented in Figure 4.

### 3.3. Cystatin C and β2-Microglobulin Correlations with Postnatal Glomerular Filtration Rate

CysC/FuCr was significantly correlated with eGFR on both day 4 (*p* = 0.01) and day 30 (*p* = 0.03) of postnatal life. Similarly, B2M/FuCr showed significant correlations with eGFR on day 4 (*p* = 0.003) and day 30 (*p* = 0.01). NGAL/FuCr correlated with eGFR on day 4; however, no significant association was found with eGFR on day 30.

In neonates who survived 30 days without renal dysfunction, significant negative correlations were observed between eGFR and all studied biomarkers on day 4: NGAL/FuCr (r = −0.67, *p* = 0.03), CysC/FuCr (r = −0.78, *p* = 0.008), and B2M/FuCr (r = −0.85, *p* = 0.002). On day 30, eGFR remained significantly correlated with CysC/FuCr (r = −0.82, *p* = 0.006) and B2M/FuCr (r = −0.86, *p* = 0.002).

In contrast, no significant correlations between eGFR (on either day 4 or day 30) and the studied biomarkers were observed in neonates who developed renal dysfunction by day 30 or in those who died within 30 days after birth.

**Figure 5:**
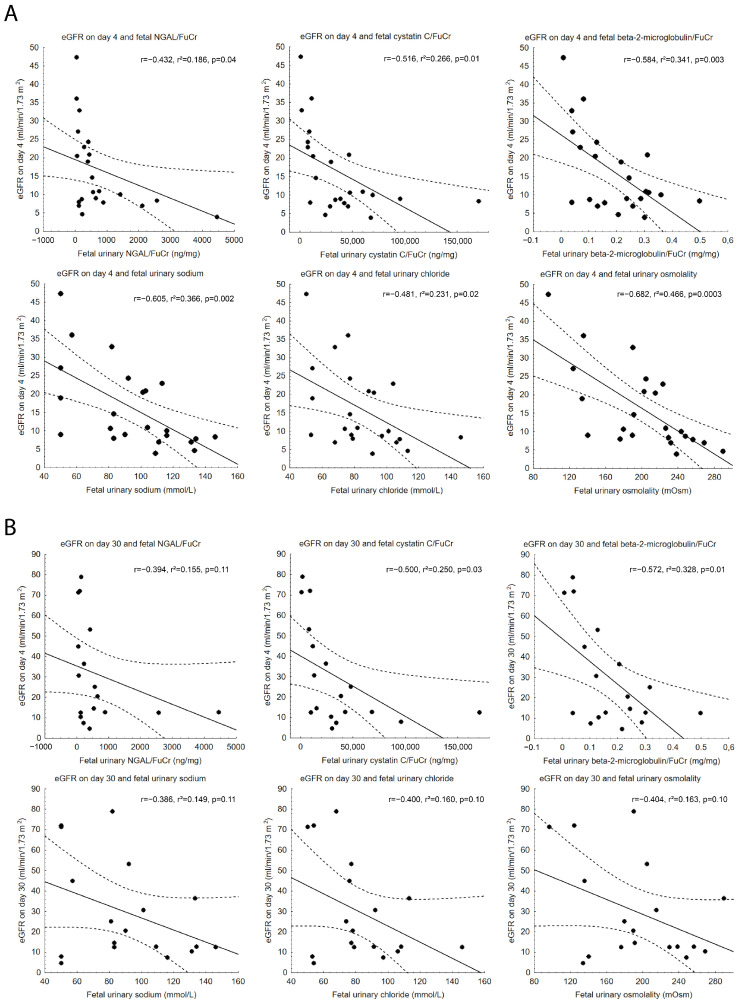
Scatter plots presenting correlations between fetal urinary biomarkers and renal function, defined as eGFR on days 4 and 30 of postnatal life. (**A**) Scatter plots presenting correlations with eGFR on day 4. (**B**) Scatter plots presenting correlations with eGFR on day 30. Regression lines represent 95% confidence interval. eGFR—estimated glomerular filtration rate; FuCr—fetal urinary creatinine; NGAL—neutrophil gelatinase-associated lipocalin.

Among conventional biomarkers, fetal urinary sodium correlated with eGFR on day 4 (*p* = 0.002), whereas no correlation with eGFR on day 30 has been found (*p* = 0.11). Similarly, fetal urinary chloride (*p* = 0.02) and osmolality (*p* = 0.0003) correlated only with eGFR assessed on day 4. Correlations of novel and conventional biomarkers with eGFR are presented in Figure 5.

## 4. Discussion

The main findings of our study are as follows: (1) Fetal urinary NGAL, CysC and B2M predict composite endpoint occurrence defined as impaired renal function and/or mortality within 30 days after birth in fetuses in LUTO; (2) fetal urinary CysC and B2M negatively correlate with postnatal GFR only in fetuses with normal renal function on day 30 after birth.

Adequate prenatal prediction of postnatal renal dysfunction and mortality is crucial for both pre- and postnatal counseling. In recent years, several studies have aimed to improve the prediction of poor postnatal renal function [12,14,15,16]. A study by Klein et al. [14] identified and validated 12 fetal urinary peptides, which predicted postnatal renal function in patients with PUV as a composite indicator called 12PUV. The 12PUV classifier identified patients who developed end-stage renal disease within 2 years after birth with good diagnostic performance (AUC 0.94, 95%CI 0.82–0.99), a sensitivity of 88% (95%CI 66–98%) and a specificity of 95% (95%CI 80–100%). It should be noted that despite high diagnostic performance, limited availability and a relatively high cost of protein laboratory measurements limit potential clinical applications of 12PUV. Comparable results were also confirmed in an independent, single-center observational study published by Buffin-Meyer et al. [12]. One study evaluated inflammatory proteins obtained from fetal urine as predictors of postnatal renal function. Three fetal urinary chemokines, CCL2, CCL4 and CXCL9, predicted postnatal kidney failure with AUCs of 0.87, 0.86 and 0.81, respectively. Composite indicator including all three chemokines reached comparable predictive performance at 12PUV; however, validation of this tool in further studies is required [15].

Our study identified the performance of NGAL, CysC and B2M in the prediction of adverse outcomes such as postnatal renal function impairment on postnatal day 30 or death within the first 30 days of life (assessed as a composite endpoint). Acceptable predictive performance was observed for fetal urinary CysC with an AUC of 0.857 (95% CI: 0.7–1.0), whereas fair diagnostic performance was observed for both NGAL and B2M: 0.793 (95% CI: 0.614–0.972 and 0.764 (95% CI: 0.562–0.966), respectively.

The role of NGAL, CysC and B2M in the prediction of renal dysfunction and/or death seems to be associated with the enhanced output of these biomarkers in tubular injury, which may occur in fetuses suffering from LUTO [2].

In recent years, several studies have evaluated the role of both serum and urinary measurements of CysC, NGAL and B2M [2]. Urinary NGAL has been found to correlate with eGFR. A recently published study by Srivastava et al. [17] showed significantly elevated urinary NGAL levels in pediatric patients with neurogenic bladder, especially in those with decreased eGFR and kidney scarring [17]. Moreover, several studies have demonstrated increased formation of NGAL shortly after tubular injury, especially within distal tubules [18,19]. In our population, LUTO may result in impaired urine outflow, which may lead to both functional and structural damage in fetal kidneys.

Physiologically, the urinary concentration of B2M remains minimal; however, after tubular injury, its concentration rises notably due to impaired reabsorption [20]. Previous studies have shown a correlation between urinary B2M, inflammatory biomarkers potentially involved in kidney damage, and a decreased number of glomeruli [2,21]. Among fetuses with PUV, a study by Vieira et al. [2] showed significantly higher levels of fetal urinary B2M when compared to controls. B2M positively correlated with inflammatory biomarkers (interleukin-8, eotaxin, interferon-γ-inducible protein-10, monocyte chemotactic protein 1 and soluble TNF receptor 1), which are shown to be related to unfavorable outcomes such as renal dysfunction and death [2,6,7]. Therefore, increased urinary B2M in fetuses with LUTO may indicate more advanced kidney damage.

Compared to NGAL and B2M, in physiological conditions, only small amounts of CysC can be found in urine, as it undergoes approximately complete metabolism within proximal tubules. In case of tubular damage, tubular metabolism may be impaired, which results in increased CysC urine output [10].

Therefore, renal response to urinary tract obstruction remains complex and involves inflammatory and profibrotic pathways activation leading to functional and structural damage [2,6,7,22]. According to the results of our study, levels of fetal urinary NGAL, CysC and B2M may reflect prenatal kidney dysfunction related to LUTO at an early stage, along with the severity of clinical conditions; however, direct correlation between eGFR and was reached only for CysC and B2M, and only among fetuses with preserved renal function on day 30 after birth. This finding may suggest that the studied biomarkers are particularly informative during earlier or less advanced stages of renal injury, when nephron function is still partially preserved, and the relationship between biomarker expression and eGFR remains linear. In contrast, in fetuses with adverse outcomes (renal dysfunction or death), disrupted nephrogenesis and progressive structural damage may lead to non-linear or heterogeneous eGFR patterns, thereby masking direct correlations. This observation suggests that the predictive value of presented biomarkers is rather stage-dependent, whereas their biological significance remains unresolved. Moreover, it remains to be determined whether the studied biomarkers may be particularly useful in identifying fetuses at risk of adverse outcomes, especially prior to the development of irreversible kidney damage.

The issues that need to be addressed are potentially changing urinary outputs of NGAL, CysC and B2M during ongoing development and maturation of renal tubules. To date, data on potential changes in NGAL, CysC and B2M concentrations in fetal and neonatal urine remain scarce. In our population, we observed a stronger correlation between CysC, B2M and eGFR when compared to NGAL. Moreover, NGAL did not correlate with eGFR on day 30th of postnatal life, whereas CysC and B2M did. Apart from several methodological explanations for this phenomenon (such as the small number of patients included in this study, along with potential outliers), the significant role of NGAL in the development and maturation of renal tubules should be considered [23]. Although NGAL is released due to ongoing deciliation secondary to tubular injury, it may have a role in tubular maturation. In postnatal life, further differentiation and proliferation of tubular cells occur secondarily to stimuli from mechanosensors, which are sensitive to increased tubular flow [24]. This raises a question regarding whether shear stress in renal tubules may lead to increased expression of NGAL in healthy kidneys and if this mechanism could be impaired due to changes secondary to LUTO. Serial pre- and postnatal measurements of NGAL in patients with LUTO may be required to evaluate potential changes affecting the interpretation and applicability of NGAL as a biomarker of kidney dysfunction in patients with LUTO. To summarize, currently available data indicate potential involvement of NGAL in tubular development, which is not the case in CysC and B2M. Therefore, CysC and B2M may potentially better reflect long-term renal function postnatally, as their expression might be less affected by physiologically ongoing developmental processes.

Our study is not without limitations. The main limitation is the limited number of subjects and the single-center study design. The limited number of primary endpoints (death and/or postnatal renal failure) did not allow us to conduct analyses separately. Thus, a composite endpoint composed of deaths and/or renal failure has been created. It should be noted that postnatal deaths may not be only related to renal dysfunction secondary to LUTO, as, specifically, all subjects were pre-term neonates with a median birth age of 34.50 weeks (IQR: 33.0–37.0 weeks). Therefore, postnatal deaths might have been related to prematurity complications. Another limitation is the method of eGFR estimation based on sCr. SCr may be prone to deviations due to several factors that influence its concentration. Unfortunately, the study did not allow us to understand the mechanisms of death. Finally, a small sample did not allow us to form a multivariable model to predict unfavorable outcomes. Moreover, it should be noted that based on ROC analysis, all novel biomarkers reached statistical significance in the prediction of composite endpoints, whereas in the analysis based on logistic regression, NGAL did not. These results are probably related to the limitations of statistical analysis, as ROC analysis is less sensitive to distributional assumptions and can detect predictive values even when the relationship is non-linear or when the effect is concentrated at extreme values. Univariate logistic regression may fail to reach significance if the relationship is not linear, if there is substantial overlap in biomarker values between outcome groups, or if the sample size is limited, reducing statistical power.

Assuming that the small cohort in our study was a potentially confounding variable, this may have affected the results presented in this manuscript. We have described this observation in the limitations discussed above.

Nevertheless, the results of our study indicate the potential role of novel biomarkers obtained from fetal urine (NGAL, CysC, B2M) in the prediction of renal dysfunction or death within 30 days after birth, which seem to be superior to classical ones (fetal urine output of Na^+^, Cl^-^ and osmolality). Finally, significant correlations between novel fetal urinary biomarkers and postnatal kidney function were found. To overcome the limitations of our study, further, large-scale studies are required to (i) establish the role of NGAL, CysC and B2M in the prediction of renal dysfunction development (preferably using more accurate endpoints than decreased eGFR such as renal scintigraphy, serum CysC measurements or need for renal replacement therapy) and (ii) assess the role of novel biomarkers in kidney development to better understand their potential influence on pre- and postnatal renal development.

Finally, considering the limitations of the present study, the clinical applicability of these biomarkers remains to be established. Further studies are required before they can be implemented in clinical practice.

## 5. Conclusions

In conclusion, fetal urinary NGAL, CysC and B2M constitute a promising tool in early prediction of adverse clinical outcomes such as impaired renal function and mortality within 30 days after birth in fetuses with LUTO. Moreover, CysC and B2M may predict postnatal eGFR in fetuses with LUTO. It seems that multifactorial kidney damage, which occurs in LUTO due to abnormal urine outflow, which also involves renal tubules, may be represented by fetal urinary NGAL, CysC and B2M. However, due to the lack of data and several inconclusive observations, none of the presented biomarkers are currently applicable in clinical decision making. Further, multicenter studies are crucial to validate NGAL, CysC and B2M in the prediction of adverse clinical outcomes in fetuses with LUTO, which may improve the accurate prediction of renal function decline after birth and support pre- and postnatal counseling, including prenatal intervention decision making.

## Figures and Tables

**Table 1:** Group characteristics. Numbers in 2nd–5th columns represent median (interquartile range Q1–Q3) or average ± standard deviation unless stated otherwise. Presented *p*-value represents comparison between IUFD, RD, ND and N.

	Total (*n* = 38)	IUFD (*n* = 14)	RD (*n* = 9)	ND (*n* = 5)	N (*n* = 10)	*p*
Mother age, years	29.26 ± 4.84	29.93 ± 4.92	29.67 ± 3.43	27.4 ± 3.98	28.9 ± 6.43	0.78
Timing of prenatal intervention, weeks	19.82 ± 4.37	17.93 ± 3.56	22.22 ± 3.87	18.2 ± 1.48	21.10 ± 5.60	0.07
Shunting, *n* (%)	32 (84.21)	8 (57.14)	9 (100)	5 (100)	10 (100)	0.007
Amnioinfusion, *n* (%)	17 (44.73)	6 (42.86)	6 (66.67)	2 (40.0)	3 (30.0)	0.44
Amnioreduction, *n* (%)	1 (2.63)	0 (0)	0 (0)	0 (0)	1 (10.0)	0.41
Urinary Na^+^	103.5 (83–117)	114.5 (98–123)	109.0 (83–131)	104.0 (103–111)	86.00 (57–101)	0.16
Urinary Cl^−^	87.5 ± 19.48	93.14 ± 11.90	90.11 ± 28.99	84.8 ± 11.95	78.6 ± 19.93	0.32
Urine Osmolality	212.37 ± 45.24	232.21 ± 30.51	209.0 ± 50.46	218.8 ± 21.60	184.4 ± 55.51	0.08
NGAL, ng/mL	15.55 (7.37–31.01)	15.55 (9.16–31.9)	22.58 (7.37–87.08)	26.78 (16.8–65.23)	7.78 (2.01–19.98)	0.31
CysC, ng/mL	1237.34 (456.54–2147.16)	1782.36 (692.6–2209.0)	1212.72 (884.76–2778.56)	1780.24 (1321.78–2147.16)	412.64 (223–789.84)	0.08
B2M, mg/mL	15.55 (3.64–11.35)	8.0 (5.28–13.00)	7.51 (4.08–11.46)	10.94 (8.0–11.76)	3.59 (2.13–7.21)	0.32
NGAL/FuCr, ng/mg	378.9 (204.72–880.65)	358.45 (239.41–996.88)	537.62 (204.72–2561.18)	743.89 (442.11–1412.08)	171.52 (62.81–406.32)	0.19
CysC/FuCr, ng/mg	34274.50 (11124.95–59702.7)	48178.17 (17500.56–68849.84)	33686.67 (28540.65–67769.76)	46848.42 (45863.74–59643.33)	9906.69 (7193.55–23934.55)	0.08
B2M/FuCr, mg/mg	0.20 (0.11–0.30)	0.20 (0.16–0.34)	0.21 (0.13–0.29)	0.30 (0.26–0.31)	0.10 (0.04–0.2)	0.32
Prenatal ultrasound						
AFI, mm	53.5 (25–88)	35.0 (14–70)	40 (25–80)	46.4 (30–59)	95 (56–140)	0.16
MVP, mm	21.0 (3–33)	19.0 (2–24)	3 (1.5–37)	26.5 (11.5–32.5)	30 (18–42)	0.59
Keyhole sign	25 (65.79)	9 (64.29)	6 (66.67)	2 (40.0)	8 (80.0)	0.50
Megabladder	34 (89.47)	12 (85.71)	8 (88.89)	5 (100.0)	9 (90.0)	0.84
Bladder sagittal diameter, mm	43.03 ± 15.03	39.38 ± 12.61	41.11 ± 14.65	49.60 ± 20.51	46.60 ± 16.07	0.49
Bladder wall thickness, mm	2.09 ± 1.12	2.54 ± 1.04	2.98 ± 1.12	3.62 ± 0.88	2.99 ± 1.27	0.31
Hydronephrosis, *n* (%)	15 (39.47)	5 (35.71)	6 (66.67)	1 (20.0)	3 (30.0)	0.26
Unilateral, *n* (%) of total hydronephrosis	3 (7.89)	0 (0.0)	3 (33.33)	0 (0)	0 (0)	0.01
Kidney hyperechogenicity, *n* (%)	23 (60.53)	7 (50.0)	7 (77.78)	3 (60.0)	6 (60.0)	0.84
Kidney cysts, *n* (%)	8 (21.05)	2 (14.29)	2 (22.22)	2 (40.0)	2 (20.0)	0.76
Right kidney						
AP diam. mm	19.14 ± 8.45	16.12 ± 7.85	20.31 ± 5.62	20.67 ± 8.08	21.17 ± 11.5	0.55
Transverse diam. mm	15.7 ± 7.05	12.66 ± 6.37	17.1 ± 5.34	18.23 ± 6.93	17.17 ± 9.04	0.38
Right kidney long diam. mm	27.95 ± 12.07	21.56 ± 10.53	33.98 ± 9.63	33.0 ± 12.49	28.06 ± 13.07	0.11
RPD, mm	6.5 (1.7–12.0)	1.8 (1.2–8.0)	8.0 (7–14)	3.8 (1.5–5.0)	6.0 (4.0–12.0)	0.02
Left kidney						
AP diam. mm	21.34 ± 11.37	17.34 ± 9.27	13.56 ± 13.24	19.53 ± 6.08	21.7 ± 12.89	0.37
Transverse diam. mm	16.65 ± 7.82	12.47 ± 6.64	20.04 ± 7.24	16.13 ± 3.80	18.52 ± 9.18	0.14
Long diam. mm	27.71 ± 13.78	21.74 ± 12.38	33.11 ± 14.53	32.67 ± 13.43	27.94 ± 19.0–37.6	0.29
RPD, mm	6.4 (1.8–10.0)	2.0 (1.2–8.0)	9.0 (7.0–16.0)	5.0 (1.5–6.8)	6.0 (4.2–15.0)	0.25

AFI—amniotic fluid index, AP diam.—anteroposterior diameter, B2M—beta-2-microglobulin, CysC—Cystatin C, FuCr—fetal urinary creatinine, IUFD—intra-uterine fetal death, Long diam.—longitudinal diameter, MVP—maximum vertical pocket, ND—neonatal death, N—normal (survived 30 days without renal dysfunction on day 30 after death), RD—renal dysfunction, RPD—renal pelvic diameter.

**Table 2:** Baseline characteristics of live-born neonates with LUTO. Numbers in 2nd–4th columns represent median (interquartile range Q1–Q3) or average ± standard deviation unless stated otherwise. Presented *p*-value represents comparison between the RD or death subgroup and the normal renal function subgroup.

	Live-Born Neonates (*n* = 24)	RD + ND (*n* = 14)	N (*n* = 10)	*p*
Birth age, weeks	34.50 (33.0–37.0)	34.50 (31.0–36.0)	34.5 (33.0–38.0)	0.23
Weight, g	2589.17 ± 834.40	2395.00 ± 756.09	2861.00 ± 901.52	0.18
Length, cm	47.17 ± 6.43	46.07 ± 6.83	48.89 ± 5.69	0.31
Head circumference, cm	32.00 (30.0–33.0)	32.00 (30.0–33.0)	32.00 (31.0–33.0)	0.73
APGAR 1st minute	8.00 (6.0–9.0)	7.50 (6.0–9.0)	9.00 (8.0–9.0)	0.11
Lung hypoplasia, *n* (%)	6 (25.00)	5 (35.71)	1 (10.00)	0.34

APGAR 1st minute—appearance pulse grimace activity respiration (score at 1 min after birth), ND—neonatal death, N—normal (survived 30 days without renal dysfunction on day 30 after death), RD—renal dysfunction.

**Table 3:** Exploratory descriptive data showing thresholds and their parameters, including Youden index, sensitivity and specificity for NGAL, cystatin C, β2-microglobulin and their corrected for FuCr values in prediction of renal dysfunction and death within 30 days after birth. It should be noted that, due to several limitations, presented thresholds are not applicable in clinical practice (see limitations in Section 4, Discussion).

	Threshold	Youden Index	Sensitivity	Specificity	AUC
Prediction of composite endpoint					
Novel biomarkers					
NGAL	26.78 ng/mL	0.50	0.50	1.00	0.786
Cystatin C	456.54 ng/mL	0.70	1.00	0.70	0.879
β2-microglobulin	3.49 mg/mL	0.47	0.93	0.50	0.779
NGAL/FuCr	110.32 ng/mg	0.50	1.00	0.40	0.793
Cystatin C/FuCr	14763.87 ng/mg	0.63	1.00	0.40	0.857
β2-microglobulin/FuCr	0.13 mg/mg	0.49	0.79	0.70	0.764
Conventional biomarkers					
Urinary Na^+^	E: 103 mmol/L N: 100 mmol/L	E: 0.44 N: 0.34	E: 0.64 N: 0.64	E: 0.80 N: 0.70	0.693
Urinary Cl^−^	E: 79 mmol/L N: 90 mmol/L	E: 0.41 N: 0.16	E: 0.71 N: 0.46	E: 0.70 N: 0.70	0.639
Osmolality	E: 226 mOsm N: 200 mOsm	E: 0.47 N: 0.26	E: 0.57 N: 0.66	E: 0.90 N: 0.60	0.700
Prediction of renal dysfunction assessed on day 30. after birth					
Novel biomarkers					
NGAL	22.58	0.46	0.56	0.90	0.767
Cystatin C	456.54 ng/mL	0.70	1.00	0.70	0.856
β2-microglobulin	3.64	0.39	0.89	0.50	0.733
NGAL/FuCr	880.65	0.44	0.44	1.00	0.756
Cystatin C/FuCr	15144.76	0.59	0.89	0.70	0.833
β2-microglobulin/FuCr	0.13 mg/mg	0.49	0.78	0.70	0.722
Conventional biomarkers					
Urinary Na^+^	E: 109 mmol/L N: 100 mmol/L	E: 0.36 N: 0.34	E: 0.56 N: 0.64	E: 0.80 N: 0.70	0.644
Urinary Cl^−^	E: 79 mmol/L N: 90 mmol/L	E: 0.37 N: 0.16	E: 0.67 N: 0.46	E: 0.70 N: 0.70	0.664
Osmolality	E: 229 mOsm N: 200 mOsm	E: 0.46 N: 0.26	E: 0.56 N: 0.66	E: 0.90 N: 0.60	0.656

E represents estimated value based on our cohort, whereas N represents calculations for widely applied threshold for conventional biomarkers. AUC—area under the receiver operator characteristic (ROC) curve; FuCr—fetal urinary creatinine; NGAL—neutrophil gelatinase-associated lipocalin.

## Data Availability

The data presented in this study are available on request from the corresponding author.

## Supplementary Materials

The following supporting information can be downloaded at: https://www.mdpi.com/article/10.3390/jcm15052056/s1, Table S1: Comparison of live-born neonates. Two subgroups were compared: neonates who died or developed renal dysfunction (Composite endpoint) and those with normal renal function. Numbers in second, third and fourth column represent median (interquartile range Q1–Q3) or average ± standard deviation unless stated otherwise.

## Abbreviations

The following abbreviations are used in this manuscript:

AFI	Amniotic fluid index
AP	Anterior–posterior
AUC	Area under the receiver operating characteristic curve
B2M	β2-microglobulin
CI	Confidence interval
CysC	Cystatin C
Diam.	Diameter
eGFR	Estimated glomerular filtration rate
FuCr	Fetal urinary creatinine
IQR	Interquartile range
LUTO	Lower urinary tract obstruction
MVP	Maximum vertical pocket
NGAL	Neutrophil gelatinase-associated lipocalin
PUV	Posterior urethral valve
RD	Renal dysfunction
ROC	Receiver operating characteristic curve
RPD	Renal pelvic diameter
sCr	Serum creatinine
TNF	Tumor necrosis factor
