# Supplementary material for: Fetal Urinary Cystatin C, NGAL and Beta-2-Microglobulin as Predictors of Postnatal Renal Function Impairment and Death in Fetuses with Lower Urinary Tract Obstruction
Source: J Clin Med. 2026 Mar 8;15(5):2056. doi: 10.3390/jcm15052056 (PMC12986259; doi:10.3390/jcm15052056)
Supplement: Supplementary file 1 [file jcm-15-02056-s001.zip › jcm-4125643-supplementary.pdf]

Table S1. Comparison of born-alive neonates. Two subgroups were compared: neonates who died or developed renal dysfunction (Composite endpoint) and those with normal renal function.

	**Total (n=24)**	**RD or death (n=14)**	**Normal renal function (n=10)**	**p**
Mother age, years	28.88 ± 4.86	28.86 ± 3.66	28.90 ± 6.40	0.98
Timing of prenatal intervention, weeks	20.92 ± 4.49	20.79 ± 3.72	21.10 ± 5.61	0.87
Shunting, n (%)	24 (100)	14 (100)	10 (100.00)	1.00
Amnioinfusion, n (%)	11 (45.83)	8 (57.14)	3 (30.00)	0.24
Amnioreduction, n (%)	1 (4.17)	0 (0.00)	1 (10.00)	0.42
**Prenatal ultrasound**				
AFI, mm	7.37 ± 5.65	5.24 ± 4.07	10.34 ± 6.38	0.02
MVP, mm	22.21 ± 18.99	16.45 ± 16.88	29.70 ± 19.79	0.10
Keyhole sign	16 (66.67)	8 (57.14)	8 (80.00)	0.39
Megabladder	22 (91.67)	13 (92.86)	9 (90.00)	1.00
Bladder sagittal diameter, mm	45.17 ± 16.14	44.14 ± 16.71	46.6 ± 16.07	0.72
Bladder wall thickness, mm	3.12 ± 1.13	3.21 ± 1.05	2.99 ± 1.27	0.65
Hydronephrosis, n (%)	10 (41.67)	7 (50.00)	3 (30.00)	0.42
Unilateral, n (%) of total hydronephrosis	3 (30.00)	3 (43.86)	0 (0.00)	0.24
Kidney hyperechogenicity, n (%)	16 (66.67)	10 (71.43)	6 (60.00)	0.67
Kidney cysts, n (%)	6 (25.00)	4 (28.57)	2 (20.00)	1.00
**Right kidney**				
AP diam. mm	20.73 ± 8.50	20.4 ± 5.91	21.17 ± 11.50	0.84
Transverse diam. mm	17.29 ± 7.00	17.38 ± 5.45	17.17 ± 9.04	0.95
Right kidney long diam. mm	31.30 ± 11.67	33.73 ± 9.80	28.06 ± 13.71	0.28
RPD, mm	7.00 (4.0-12.0)	7.0 (4.40-13.0)	6.0 (4.0-12.0)	0.86
**Left kidney**				
AP diam. mm	23.43 ± 12.01	24.73 ± 11.71	21.70 ± 12.89	0.58
Transverse diam. mm	18.83 ± 7.61	19.07 ± 6.63	18.52 ± 9.18	0.88
Long diam. mm	30.83 ± 13.70	33.00 ± 13.65	27.94 ± 14.02	0.42
RPD, mm	7.00 (5.0-15.0)	7.50 (5.0-13.0)	6.0 (4.2-15.0)	0.62
**Neonate**				
Birth age, weeks	34.50 (33.0-37.0)	34.50 (31.0-36.0)	34.5 (33.0-38.0)	0.23
Weight, g	2589.17 ± 834.40	2395.00 ± 756.09	2861.00 ± 901.52	0.18
Length, cm	47.17 ± 6.43	46.07 ± 6.83	48.89 ± 5.69	0.31
Head circumference, cm	32.00 (30.0-33.0)	32.00 (30.0-33.0)	32.00 (31.0-33.0)	0.73
APGAR 1st minute	8.00 (6.0-9.0)	7.50 (6.0-9.0)	9.00 (8.0-9.0)	0.11
Lung hypoplasia, n (%)	6 (25.00)	5 (35.71)	1 (10.00)	0.34
Congenital infection, n (%)	9 (37.50)	6 (42.86)	3 (30.00)	0.68

AFI – amniotic fluid index, AP diam. – anteroposterior diameter, APGAR 1st minute – appearance pulse grimace activity respiration (score at 1 minute after

birth), Long diam. – longitudinal diameter, MVP – maximum vertical pocket, RPD – renal pelvic diameter
